# Plasma PRPC Levels Correlate With Severity and Prognosis of Intracerebral Hemorrhage

**DOI:** 10.3389/fneur.2022.913926

**Published:** 2022-07-11

**Authors:** Xiaoyu Wu, Ming Liu, Tian Yan, Zefan Wang, Wenhua Yu, Quan Du, Wei Hu, Yongke Zheng, Zuyong Zhang, Keyi Wang, Xiaoqiao Dong

**Affiliations:** ^1^The Fourth School of Clinical Medicine, Zhejiang Chinese Medical University, Hangzhou, China; ^2^Department of Neurosurgery, Affiliated Hangzhou First People's Hospital, Zhejiang University School of Medicine, Hangzhou, China; ^3^Department of Intensive Care Unit, Affiliated Hangzhou First People's Hospital, Zhejiang University School of Medicine, Hangzhou, China; ^4^Department of Neurosurgery, Xixi Hospital Affiliated to Zhejiang Chinese Medical University, Hangzhou, China; ^5^Central Laboratory, Affiliated Hangzhou First People's Hospital, Zhejiang University School of Medicine, Hangzhou, China

**Keywords:** severity, prognosis, intracerebral hemorrhage, cellular prion protein, biomarker

## Abstract

**Background:**

Cellular prion protein (PRPC) exerts brain-protective effects. We determined the relationship between plasma PRPC levels and disease severity plus clinical outcome after acute intracerebral hemorrhage (ICH).

**Methods:**

A total of 138 ICH patients and 138 healthy controls were included in this prospective, observational study. Hematoma volume and Glasgow coma scale (GCS) score were used to assess disease severity. Glasgow outcome scale (GOS) scores of 1–3 and 4–5 at 90 days after stroke were defined as a poor outcome and good outcome, respectively. Using multivariate analysis, we discerned the relation of plasma PRPC levels to disease severity and poor outcome. The receiver operating characteristic (ROC) curve was built to evaluate the prognostic predictive capability.

**Results:**

Plasma PRPC levels in ICH patients were significantly higher than those in healthy controls (median, 4.20 vs. 2.02 ng/ml; *P* < 0.001), and were independently correlated with GCS score (*r* = −0.645, *P* < 0.001) and hematoma volume (*r* = 0.627, *P* < 0.001). Plasma PRPC levels were highly correlated with GOS score (*r* = −0.762, *P* < 0.001), and were substantially higher in patients with poor outcomes than in those with the good outcomes. Using maximum Youden index, plasma PRPC levels >3.893 ng/ml distinguished the risk of poor outcome at 90 days, with a sensitivity of 86.4% and a specificity of 65.8% (area under the curve, 0.809; 95% confidence interval (CI), 0.737–0.881, *P* < 0.001). Plasma PRPC levels >3.893 ng/ml were independently associated with a poor 90-day outcome with an odds ratio of 12.278 (95% CI, 5.101–29.554).

**Conclusion:**

Elevated plasma PRPC levels are significantly associated with disease severity and poor 90-day outcome in ICH patients, indicating that plasma PRPC may be used as a potential prognostic biomarker after ICH.

## Introduction

Spontaneous intracerebral hemorrhage (ICH) is one of the most common severe diseases, characterized by acute onset, high disability and mortality, and poor outcome (1, 2). Secondary brain injury caused by ICH is considered to be an important factor affecting the outcome of patients. The pathophysiological mechanisms involved in secondary brain injury are very complex, including inflammatory response, destruction of the blood–brain barrier, brain edema, cytotoxic reaction, and oxidative stress (3–6). During recent decades, some biomarkers related to the occurrence and development of secondary brain injury have drawn interest for aiding in the assessment of clinical severity and prognostic prediction.

The cellular prion protein (PRPC), is a small, cell-surface glycoprotein notable primarily for its critical role in the pathogenesis of the neurodegenerative disorders known as prion diseases (7). PRPC can greatly be expressed in the central nervous system and could exert beneficial effects on the recovery of brain function (8, 9). PRPCs were substantially elevated in brain tissues of rats with acute ischemic stroke (10). In experimental traumatic brain injury, plasma PRPC levels of rats were significantly increased (11). Interestingly, patients with aneurysmal subarachnoid hemorrhage had a significant elevation of plasma PRPC levels, which were closely associated with severity and prognosis (12). Thus, PRPC is hypothesized to be released from injured brain tissues through the damaged blood–brain barrier after acute brain injury, and circulating PRPC may be a potential biomarker of acute brain injury. In the present study, we aimed to explore whether plasma PRPC levels are associated with the severity and clinical outcome of ICH.

## Materials and Methods

### Study Population

In this perspective, observational study, we recruited first-ever ICH patients admitted to the Department of Neurosurgery at our hospital from January 2019 to October 2021. We required that all patients should be hospitalized within 24 h after the onset of ICH symptoms. In this study, we required that ICH should result from amyloidosis or hypertensive arteriosclerosis. We further excluded those patients with (1) age of <18 years, (2) intracerebral bleeding as a result of head trauma, venous sinus thrombosis, intracranial arteriovenous malformation, moyamoya disease, hemorrhagic transformation of ischemic stroke, intracranial tumor, or intracranial aneurysm, (3) previous history of hemorrhagic or ischemic stroke or (4) comorbidities such as severe infection within a month, autoimmune diseases and known malignancies. Meanwhile, a group of healthy volunteers, who were recruited at our hospital from January 2019 to October 2021, were selected as controls. Controls had normal routine laboratory tests, had no history of surgery, and were free of other diseases, such as hypertension, diabetes mellitus, hyperlipidemia, malignancies, infection, and stroke.

### Assessments

We collected the subjects' information, such as gender, age, previous specific diseases (such as hypertension, diabetes, and hyperlipidemia), cigarette smoking, alcohol drinking, vital signs, and medication. The clinical severity was assessed at admission using the Glasgow Coma Scale (GCS). The ICH volume was determined by the ABC/2 method (13). A noninvasive technique was performed at admission to measure systolic and diastolic blood pressures. We used the Glasgow outcome scale (GOS) at 90 days after stroke as the evaluation criterion of neurologic function and defined a GOS score of 1–3 as a poor outcome.

### Determinations

Venous blood samples of ICH patients were immediately taken upon completion of the head CT scan, and those of healthy controls were collected at entry into the study. The blood samples were then centrifuged and frozen in a −80°C freezer until final measurement. Plasma PRPC levels were measured using a commercial enzyme-linked immunosorbent assay kit according to the designated instructions, and the same experimenter completed all testing procedures, and it was ensured that the experimenter did not know the information about the participants.

### Statistical Analysis

SPSS 23.0 was used for statistical analysis. All quantitative variables were non-normally distributed and thereby were expressed as median (upper and lower quartiles). Mann–Whitney *U* rank-sum test was used for their intergroup comparisons. The categorical variables were expressed as the number of cases (percentage) and the comparison between two groups was performed by the χ2 test or Fisher's exact method. Spearman correlation coefficient analysis was utilized for the correlation test. Logistic regression analysis was done to assess the relationship between plasma PRPC levels and poor outcomes at 90 days in ICH patients, and the odds ratio (OR) value and 95% confidence interval (CI) were calculated. The receiver operating characteristic (ROC) curve was constructed to investigate the predictive value of plasma PRPC levels for poor outcomes in ICH patients, and the cut-off value was determined using the Youden method. Meanwhile, the corresponding sensitivity and specificity were generated. Areas under the ROC curves (AUCs) were compared using the *Z* test. The difference of *P* < 0.05 was defined as statistical significance.

## Results

### Patient Selection and Characteristics

**Figure 1:**
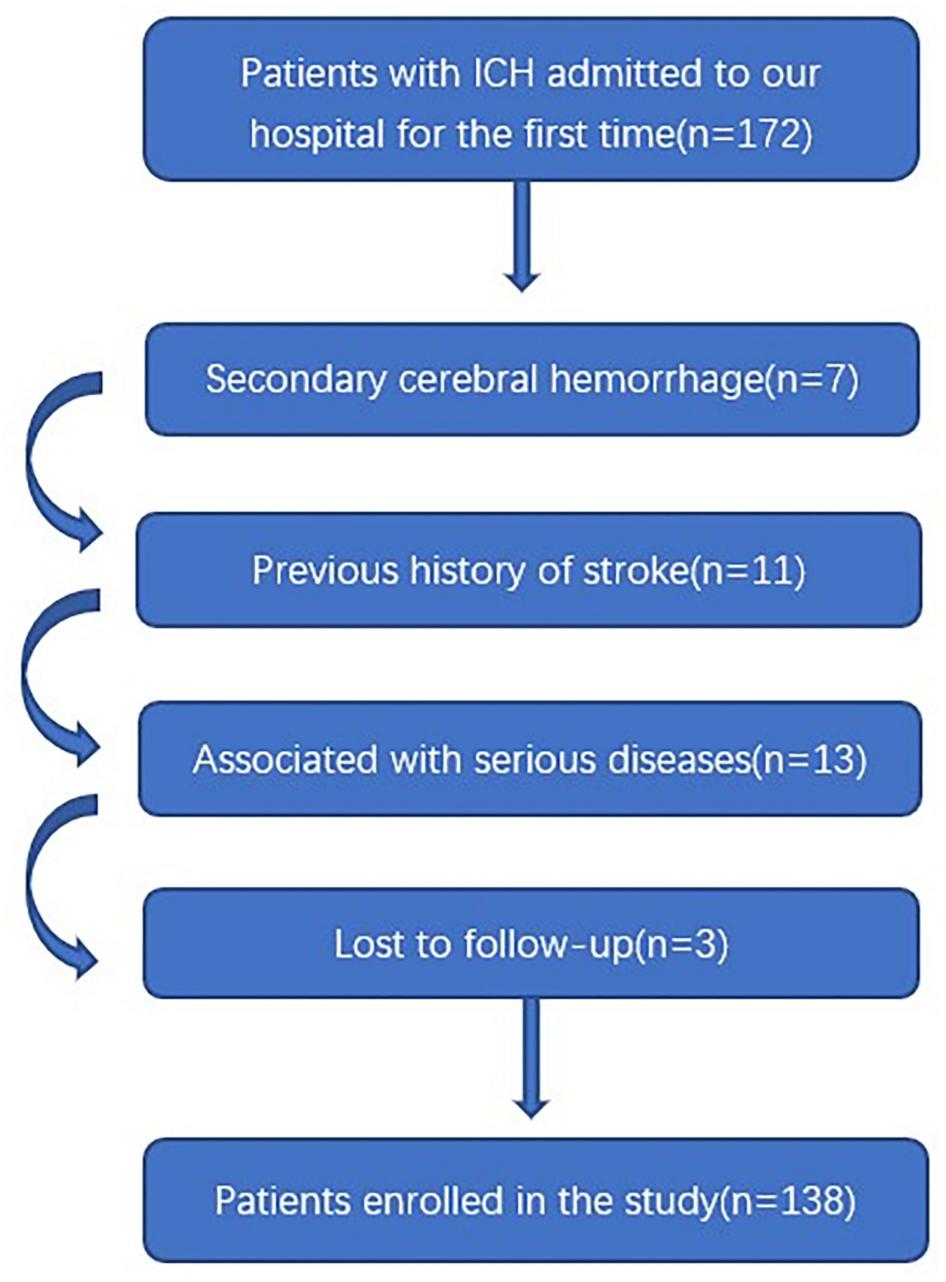
Flowing chart for screening eligible patients with intracerebral hemorrhage. ICH indicates intracerebral hemorrhage.

In this study, a total of 172 patients with first-ever ICH, who were admitted to our hospital within 24 h of onset, were initially included. Afterward, we excluded seven patients with secondary ICH, 11 patients with the previous history of stroke, 13 patients with severe diseases, and 3 patients with loss to follow-up. Finally, a total of 138 patients were enrolled in the study (Figure 1). In addition, 138 healthy controls were recruited. In Table 1, while there were no significant differences between the patients and the healthy controls in age (*P* = 0.145), sex percentage (*P* = 0.807), smoking (*P* = 0.533), and alcohol consumption (*P* = 0.130); percentages of hypertension (*P* < 0.001), diabetes mellitus (*P* < 0.001), and hyperlipidemia (*P* = 0.044) were substantially higher in the patients than in the controls.

**Table 1:** Comparisons of demographic data and vascular risk factors between controls and patients with acute intracerebral hemorrhage.

	**Patients**	**Controls**	***P*** **-value**
Age (years)	62 (52–70)	58 (50–69)	0.145
Gender (male/female)	82/56	80/58	0.807
Hypertension	82 (59.4%)	0	**<0.001**
Diabetes mellitus	18 (13.1%)	0	**<0.001**
Hyperlipidemia	4 (2.9%)	0	**0.044**
Current smoking	27 (19.6%)	23 (16.7%)	0.533
Alcohol consumption	23 (16.7%)	22 (15.9%)	0.130

*Quantitative data were reported as medians with 25th−75th percentiles. Qualitative data were presented as counts (proportions). Intergroup comparisons of various variables were performed using the χ2 test or Fisher's exact test for qualitative data, and Mann-Whitney U-test for quantitative data*.

There were 82 (59.4%) males and 56 (40.6%) females among ICH patients. Patients were aged from 23 to 89 years (median, 62 years; 25th−75th percentiles, 53–70 years). In total, 27 patients had a history of cigarette smoking and 32 patients had a history of alcohol drinking. We recorded previous medical histories, including hypertension (82 cases), diabetes (18 cases), and hyperlipidemia (4 cases). The median value of time between onset and admission was 6.7 h (range, 0.5–24 h; 25th−75th percentiles, 4–11.6 h), with their blood samples collected from 1 to 25 h (median, 7 h; 25th−75th percentiles, 4.8–12 h) after ICH. The median GCS score for severity assessment at admission was 13 (range, 4–15; 25th−75th percentiles, 9–15). According to imaging data, there were 24 patients (17.4%) with infratentorial hemorrhage and 21 patients (15.2%) with intraventricular hemorrhage. Median hematoma volume was 14.7 ml (25th−75th percentiles, 7.9–27.2 ml; range, 1.7–84.3 ml); median arterial systolic and diastolic blood pressures were 156 mmHg (range, 100–195 mmHg; 25th−75th percentiles, 140–167 mmHg) and 91 mmHg (range, 55–123 mmHg; 25th−75th percentiles, 78–100 mmHg). Laboratory examination showed a median blood leucocyte count of 8.7 × 10^9^/L (range, 3.4–20.9 × 10^9^/L; 25th−75th percentiles, 6.7–10.8 × 10^9^/L), a median blood glucose level of 6.6 mmol/L (range, 2.5–21.2 mmol/L; 25th−75th percentiles, 5.3–8.2 mmol/L) and a median blood potassium level of 3.64 mmol/L (range, 2.76–5.20 mmol/L; 25th−75th percentiles, 3.43–3.91 mmol/L). At 90 days after ICH, a total of 59 patients had a poor outcome (GOS score 1–3).

### Change of Plasma PRPC Levels

**Figure 2:**
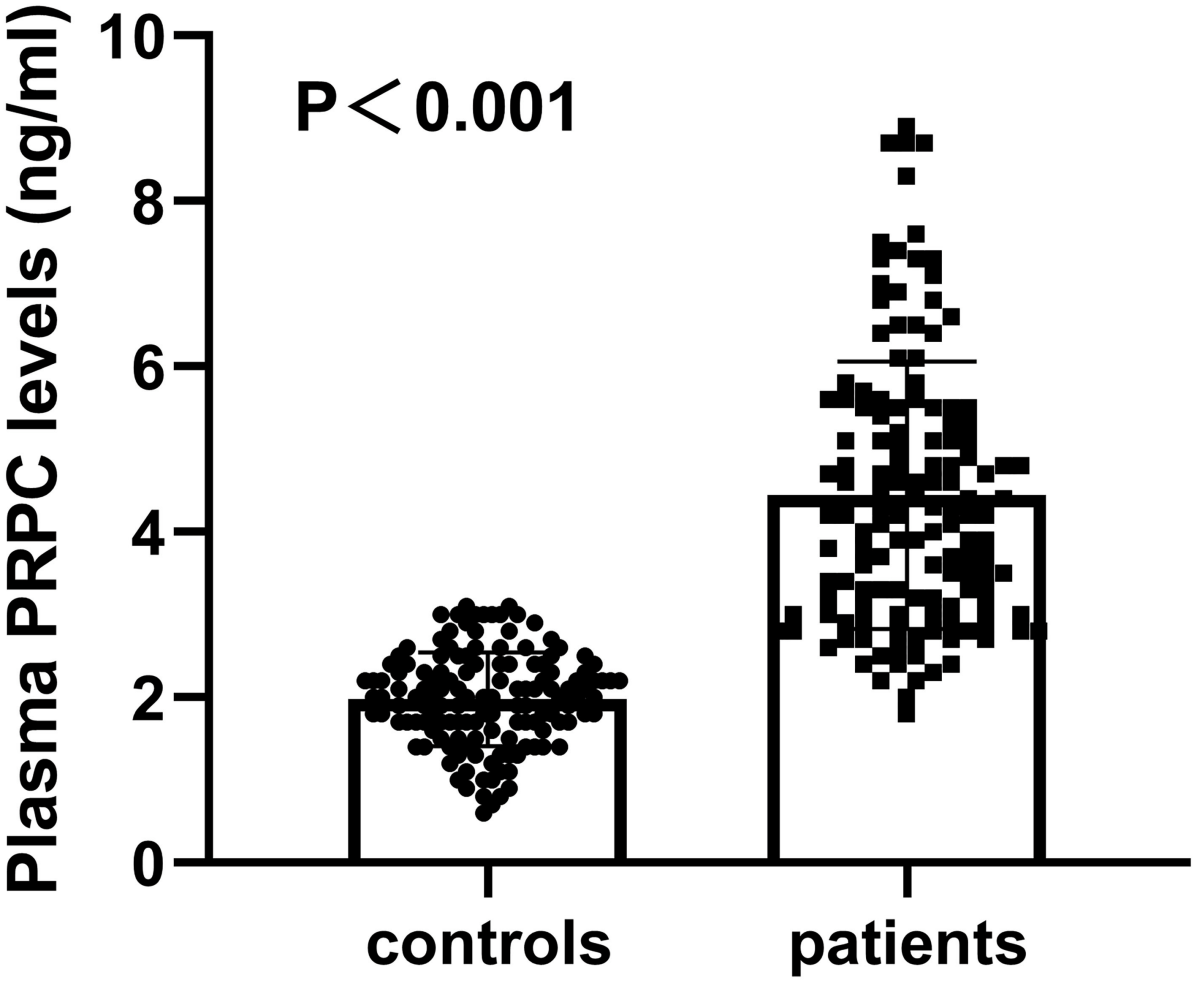
Differences in plasma cellular prion protein levels between healthy controls and patients with cerebral hemorrhage. Using the Mann–Whitney *U*-test, plasma cellular prion protein levels significantly higher in patients with ICH (*n* = 138) than in healthy controls (*n* = 138) (*P* < 0.001). Abbreviation: PRPC, cellular prion protein.

Plasma PRPC levels of ICH patients were 1.82–8.90 ng/ml, with a median of 4.20 ng/ml (25th−75th percentiles, 3.08–5.50 ng/ml). The median plasma PRPC levels in healthy controls were 2.02 ng/ml (25th−75th percentiles, 1.66–2.36 ng/ml). Using Mann–Whitney *U*-test, plasma PRPC levels were markedly higher in ICH patients than in healthy controls (*P* < 0.001, Figure 2).

### Correlation of Plasma PRPC Levels With Hemorrhagic Severity

**Figure 3:**
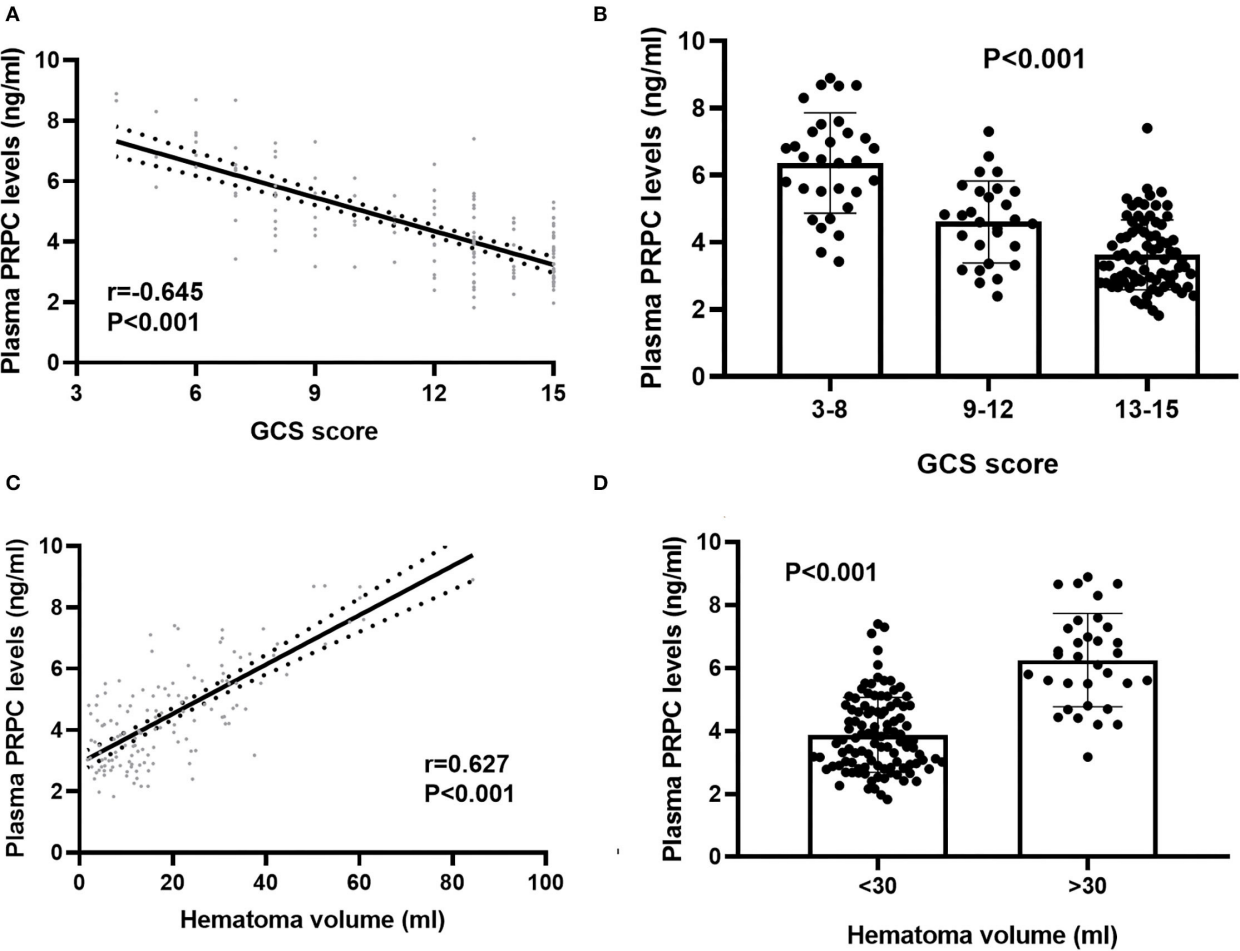
Relationship between plasma cellular prion protein levels and illness severity among intracerebral hemorrhage patients. **(A)** Correlation of plasma cellular prion protein levels with Glasgow Coma Scale score. Plasma cellular prion protein levels were significantly raised with decreasing Glasgow Coma Scale score using Spearman correlation coefficients (*r* = −0.645, *P* < 0.001). In a correlation graph, the solid line means the line of best fit, and the dashed line represents 95% confidence interval of a population mean. **(B)** Differences in plasma cellular prion protein levels among patients with different Glasgow Coma Scale scores. Glasgow Coma Scale score was identified as a categorical variable and subsequently, patients were divided into three groups in accordance with the Glasgow Coma Scale score, namely, 3–8 (*n* = 31), 9–12 (*n* = 28), and 13–15 (*n* = 79) and subsequently, patients with Glasgow Coma Scale score 3–8 had substantially highest plasma cellular prion protein levels, followed by Glasgow Coma Scale score 9–12 and then 13–15 using Kruskal–Wallis H-test (*P* < 0.001). **(C)** Correlation of plasma cellular prion protein levels with hematoma volume. Plasma cellular prion protein levels were substantially elevated with rising hematoma volume using Spearman correlation coefficients (*r* = 0.627, *P* < 0.001). In a correlation graph, the solid line means the line of best fit, and the dashed line represents 95% confidence interval of a population mean. **(D)** Differences in plasma cellular prion protein levels among patients with different hematoma volumes. Hematoma volume was identified as a categorical variable, patients with hematoma volume above 30 ml (*n* = 33) had substantially higher plasma cellular prion protein levels than those with hematoma volume below 30 ml (*n* = 105) using Mann–Whitney *U*-test (*P* < 0.001). Abbreviations: GCS, Glasgow Coma Scale; PRPC, cellular prion protein.

In order to verify the correlation of plasma PRPC levels with hemorrhagic severity indicated by GCS score and hematoma volume, GCS score and hematoma volume were defined as quantitative data. ICH patients were divided into three groups by GCS score, with scores of 3–8, 9–12, and 13–15. At the same time, 30 ml hematoma volume was taken as the critical value to classify the ICH patients, with hematoma volume >30 ml as one group, and <30ml as the other group. Subsequently, plasma PRPC levels were significantly associated with GCS score (Figures 3A,B) and hematoma volume (Figures 3C,D) of ICH patients.

Bivariate correlation analysis showed that age (*r* = 0.189, *P* = 0.026), intraventricular hemorrhage (*r* = 0.346, *P* < 0.001), hematoma volume (*r* = 0.627, *P* < 0.001), GCS score (*r* = −0.645, *P* < 0.001), and blood glucose levels (*r* = 0.250, *P* = 0.003) were highly correlated with plasma PRPC levels (Tables 2, 3). Subsequent multivariate linear regression analysis of variables with significant differences in univariate analysis demonstrated that hematoma volume and GCS score remained independently correlated with plasma PRPC levels (Table 4).

**Table 2:** Correlations between plasma cellular prion protein levels and other variables using univariate linear regression analysis in intracerebral hemorrhage.

**Components**	***t***	***P*** **-value**
Age (years)	2.172	**0.032**
Gender (male/female)	−0.427	0.670
Hypertension	1.264	0.209
Diabetes mellitus	0.423	0.673
Hyperlipidemia	−0.802	0.424
Current smoking	1.001	0.319
Alcohol consumption	−0.581	0.562
Admission time (h)	−0.951	0.343
Blood-collection time (h)	−0.919	0.360
Systolic arterial pressure (mmHg)	−1.044	0.298
Diastolic arterial pressure (mmHg)	−0.399	0.690
Infratentorial hemorrhage	−1.443	0.151
Intraventricular hemorrhage	5.591	**<0.001**
Glasgow Coma Scale score	−10.700	**<0.001**
Hematoma volume (ml)	12.746	**<0.001**
Blood leucocyte count (×10^9^/L)	1.787	0.076
Plasma glucose levels (mmol/L)	3.711	**<0.001**
Plasma potassium level (mmol/L)	−0.402	0.689

*Correlations were determined using univariate linear regression analysis*.

**Table 3:** Correlations between plasma cellular prion protein levels and other variables using bivariate correlation analysis in intracerebral hemorrhage.

**Components**	***r***	***P*** **-value**
Age (years)	0.189	**0.026**
Gender (male/female)	−0.032	0.721
Hypertension	0.093	0.277
Diabetes mellitus	−0.003	0.975
Hyperlipidemia	−0.052	0.544
Current smoking	0.083	0.336
Alcohol consumption	−0.034	0.694
Admission time (h)	−0.112	0.193
Blood-collection time (h)	−0.112	0.190
Systolic arterial pressure (mmHg)	−0.061	0.476
Diastolic arterial pressure (mmHg)	0.004	0.966
Infratentorial hemorrhage	−0.107	0.210
Intraventricular hemorrhage	0.346	**<0.001**
Glasgow Coma Scale score	−0.645	**<0.001**
Hematoma volume (ml)	0.627	**<0.001**
Blood leucocyte count (×10^9^/L)	0.158	0.064
Plasma glucose levels (mmol/L)	0.250	**0.003**
Plasma potassium level (mmol/L)	−0.059	0.495

*Correlations were determined using bivariate correlation analysis (Spearman test)*.

**Table 4:** Multivariate linear regression analysis of elevated plasma cellular prion protein levels.

**Components**	***t***	***P*** **-value**
Age (years)	1.504	0.135
Intraventricular hemorrhage	−0.563	0.575
Glasgow Coma Scale score	−2.757	**0.007**
Hematoma volume (ml)	3.328	**0.001**
Plasma glucose levels (mmol/L)	1.189	0.237

*Multivariate analysis of correlated factors was done using multivariate linear regression model*.

### Relationship Between Plasma PRPC Levels and Outcome

**Figure 4:**
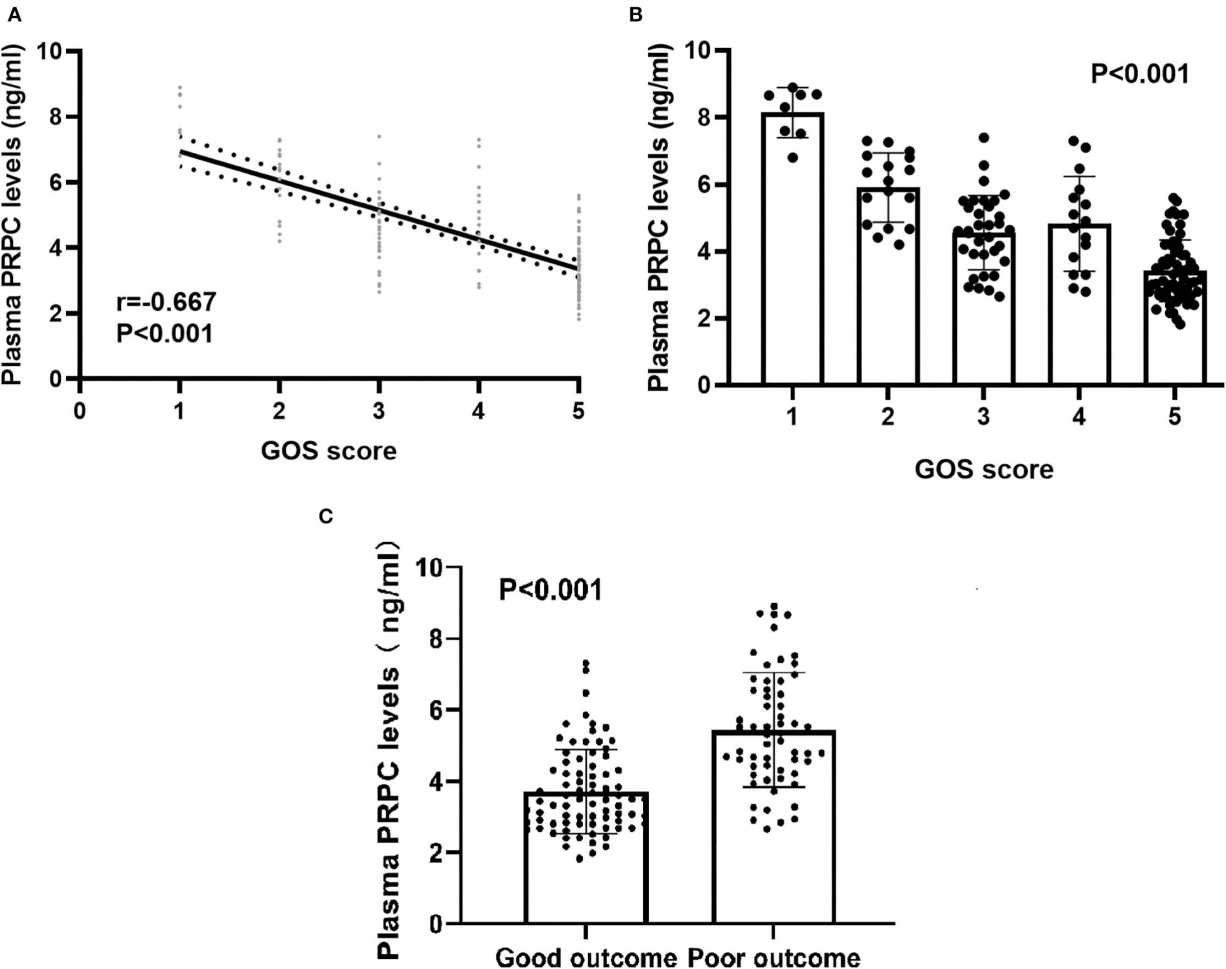
Relationship between plasma cellular prion protein levels and clinical outcome at 90 days after intracerebral hemorrhage. **(A)** Relationship between plasma cellular prion protein levels and Glasgow Outcome Scale score at 90 days after intracerebral hemorrhage. When the Glasgow Outcome Scale score was a continuous variable, plasma cellular prion protein levels were significantly declined with raised Glasgow Outcome Scale score using Spearman correlation coefficients (*r* = −0.667, *P* < 0.001). In a correlation graph, the solid line means the line of best fit, and the dashed line represents 95% confidence interval of a population mean. **(B)** Differences in plasma cellular prion protein levels among patients with Glasgow Outcome Scale score at 90 days after intracerebral hemorrhage. Plasma cellular prion protein levels significantly differed among patients with different Glasgow Outcome Scale scores using Kruskal–Wallis *H*-test (*P* < 0.001). **(C)** Comparison of plasma cellular prion protein levels between patients with good outcomes and those with poor outcomes at 90 days after intracerebral hemorrhage. Patients with a poor outcome (*n* = 59) had substantially higher plasma cellular prion protein levels than those with a good outcome (*n* = 79) using Mann–Whitney *U*-test (*P* < 0.001). Abbreviations: GOS, Glasgow Outcome Scale; PRPC, cellular prion protein.

**Figure 5:**
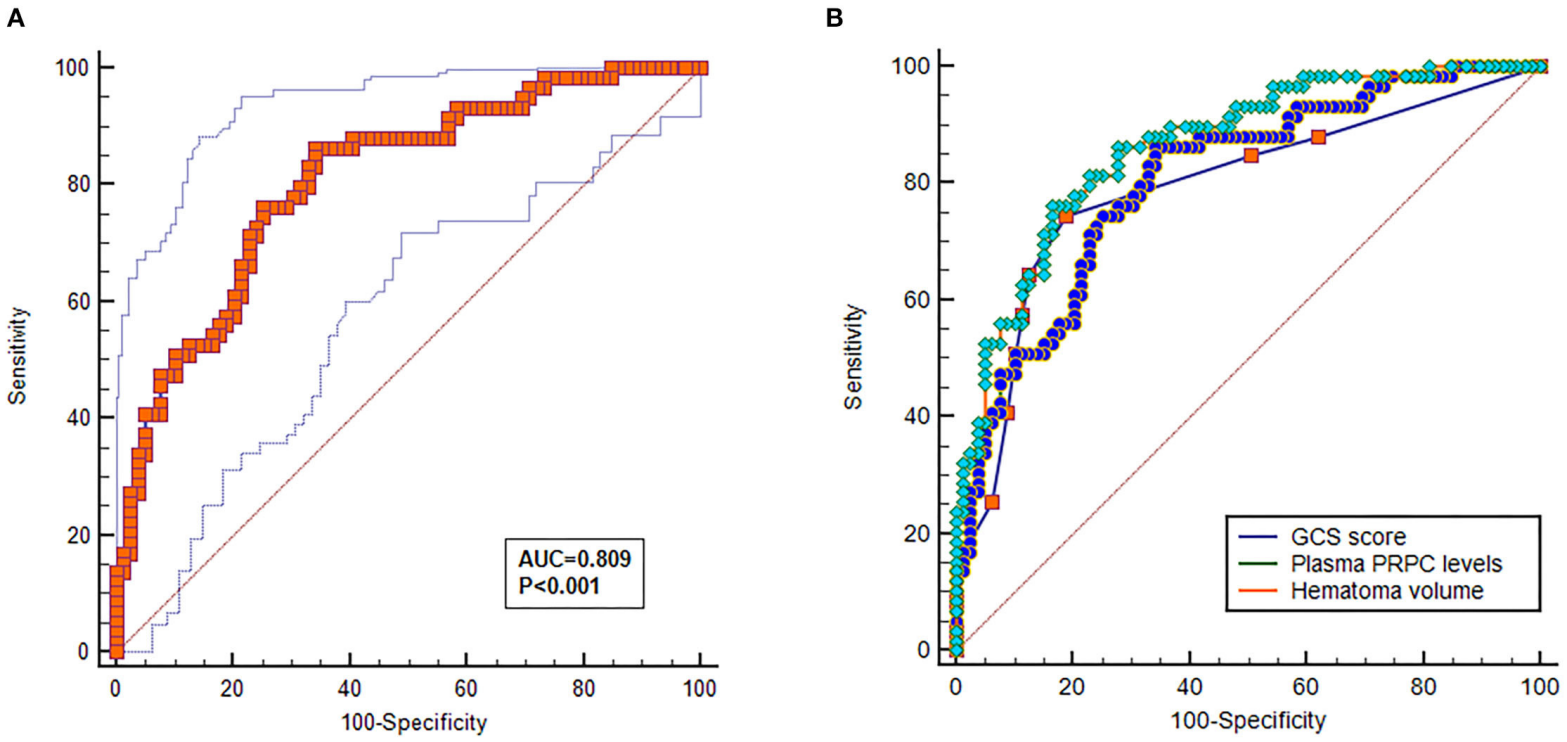
Predictive value of plasma cellular prion protein levels for poor outcome at 90 days after intracerebral hemorrhage. **(A)** Discriminatory ability of plasma cellular prion protein levels for poor outcome at 90 days after intracerebral hemorrhage. Under the receiver-operating characteristic curve, plasma cellular prion protein levels remarkably predicted the poor outcome at 90 days after intracerebral hemorrhage (area under the curve, 0.809; 95% confidence interval, 0.737–0.881); and plasma cellular prion protein levels more than 3.893 ng/ml distinguished patients with development of 90-day poor outcome with 86.4% specificity and 65.8% sensitivity, (Youden index J, 0.552). **(B)** Comparison of discriminatory capability with respect to plasma cellular prion protein levels, Glasgow Coma Scale score, and hematoma volume for 90-day poor outcome following acute intracerebral hemorrhage. Under receiver operating characteristic curve, prognostic predictive ability of plasma cellular prion protein levels (area under curve, 0.809; 95% confidence interval, 0.737–0.881) was similar to those of Glasgow Coma Scale score (area under curve, 0.793; 95% confidence interval, 0.716–0.857; *P* = 0.6462) and hematoma volume (area under curve, 0.863; 95% confidence interval, 0.795–0.916; *P* = 0.1062). Abbreviations: GOS, Glasgow Outcome Scale; PRPC, cellular prion protein.

As shown in Figures 4A,B, plasma PRPC levels showed a significant downward trend when patients' GOS scores increased. In Figure 4C, plasma PRPC levels were significantly lower in patients with good outcomes than in those with poor outcomes. In addition, the ROC curve showed that plasma PRPC levels significantly predicted poor outcomes at 90 days after ICH (AUC, 0.809; 95% CI, 0.737–0.881), and plasma PRPC levels of 3.896 ng/ml was the cutoff value, yielding the corresponding sensitivity and specificity values of 86.4 and 65.8% (Maximum Youden index J, 0.522), respectively (Figure 5A). Intriguingly, Figure 5B showed that its discriminatory ability for poor outcome was equivalent to those of GCS score (AUC, 0.793; 95% CI, 0.716–0.857; *P* = 0.6462) and hematoma volume (AUC, 0.863; 95% CI, 0.795–0.916; *P* = 0.1062).

There was a significantly higher percentage of cases with plasma PRPC levels above 3.893 ng/ml in patients with poor outcomes (GOS score 1–3) than in those with good outcomes (GOS score 4–5). In addition, as compared to patients with good outcomes, those with poor outcomes had significantly higher hematoma volume, blood leukocyte count, and glucose levels, as well as displayed substantially lower GCS scores (Tables 5, 6). Those above-mentioned variables, which were significant in univariate analysis, were included in the multivariable model, and subsequently, it was revealed that the independent predictors for poor outcome at 90 days after ICH were hematoma volume, GCS score, and plasma PRPC levels higher than 3.893 ng/ml (Table 7).

**Table 5:** Demographic, clinical, radiological and biochemical factors for 90-day poor outcome after acute intracerebral hemorrhage.

**Components**	**90-day poor outcome**		***P*** **-value**
	**Presence**	**Absence**	
Number	59 (42.8%)	79 (57.2%)	
Age (years)	63 (59–66)	61 (58–64)	0.485
Gender (male/female)	35/24	47/32	0.561
Hypertension	38 (64.4%)	44 (55.6%)	0.196
Diabetes mellitus	9 (15.3%)	9 (11.4%)	0.338
Hyperlipidemia	1 (1.7%)	3 (3.8%)	0.426
Current smoking	10 (16.9%)	17 (21.5%)	0.328
Alcohol consumption	9 (15.3%)	14 (17.7%)	0.701
Admission time (h)	6.9 (3.9–11.6)	6.3 (4.0–11.5)	0.503
Blood-collection time (h)	9.4 (7.8–11.0)	8.8 (7.4–10.3)	0.479
Systolic arterial pressure (mmHg)	151 (146–157)	154 (149–158)	0.585
Diastolic arterial pressure (mmHg)	89 (85–93)	90 (87–93)	0.954
Infratentorial hemorrhage	9 (15.3%)	15 (18.9%)	0.568
Intraventricular hemorrhage	13 (22.0%)	8 (10.1%)	0.055
Glasgow Coma Scale score	9 (4–15)	14 (6–15)	**<0.001**
Hematoma volume (ml)	28.3 (25.0–33.3)	11.4 (9.6–13.2)	**<0.001**
Blood leucocyte count ( ×10^9^/L)	9.9 (8.8–11.1)	7.9 (7.3–8.4)	**0.026**
Plasma glucose levels (mmol/L)	7.9 (7.0–8.7)	6.6 (6.1–7.2)	**0.003**
Plasma potassium level (mmol/L)	3.68 (3.55–3.80)	3.67 (3.59–3.75)	0.724
Plasma PRPC >3.893ng/ml	51 (86.4%)	27 (34.2%)	**<0.001**

*Quantitative data were reported as medians with 25th−75th percentiles. Qualitative data were presented as counts (proportions). Intergroup comparisons of various variables were performed using the χ2 test or Fisher's exact test for qualitative data, and Mann-Whitney U-test for quantitative data. Glasgow Outcome Scale score of 1–3 was designated as poor outcome. PRPC indicates cellular prion protein*.

**Table 6:** Predictive factors of 90-day poor outcome among patients with intracerebral hemorrhage using univariable logistic regression analysis.

**Components**	**Odds ratio**	**95% CI**	***P*** **-value**
Age (years)	0.997	0.972–1.023	0.833
Gender (male/female)	0.993	0.500–1.973	0.984
Hypertension	0.695	0.347–1.390	0.303
Diabetes mellitus	0.714	0.265–1.927	0.506
Hyperlipidemia	2.289	0.232–22.582	0.478
Current smoking	1.344	0.565–3.195	0.504
Alcohol consumption	1.197	0.479–2.987	0.701
Admission time (h)	1.015	0.961–1.072	0.592
Blood-collection time (h)	1.015	0.962–1.070	0.589
Systolic arterial pressure (mmHg)	0.993	0.977–1.010	0.428
Diastolic arterial pressure (mmHg)	0.998	0.976–1.022	0.891
Infratentorial hemorrhage	0.768	0.311–1.899	0.568
Intraventricular hemorrhage	2.508	0.965–6.533	0.059
Glasgow Coma Scale score	0.670	0.580–0.774	**<0.001**
Hematoma volume (ml)	1.153	1.099–1.211	**<0.001**
Blood leucocyte count ( ×10^9^/L)	1.113	1.012–1.268	**0.031**
Plasma glucose levels (mmol/L)	1.182	1.029–1.358	**0.018**
Plasma potassium level (mmol/L)	1.032	0.463–2.304	0.938
Plasma PRPC >3.893 ng/ml	12.278	5.101–29.554	**<0.001**

*Correlations were determined using bivariate correlation analysis (Spearman test) and univariate linear regression analysis. 95% CI indicates 95% confidence interval; PRPC, cellular prion protein*.

**Table 7:** Multivariate linear regression analysis of elevated plasma cellular prion protein levels.

**Variables**	**Odds ratio (95% CI)**	***P*** **-value**
Glasgow Coma Scale score	0.763 (0.592–0.992)	**0.039**
Hematoma volume (ml)	1.219 (1.105–1.345)	**<0.001**
Blood leucocyte count ( ×10^9^/L)	1.118 (0.955–1.309)	0.167
Plasma PRPC >3.893 ng/ml	4.840 (1.620–14.464)	**0.005**
Plasma glucose levels (mmol/L)	1.131 (0.916–1.396)	0.253

*Results were presented as odds ratio (95% confidence interval) using the binary logistic regression analysis. 95% CI indicates 95% confidence interval; PRPC, cellular prion protein*.

## Discussion

To the best of our knowledge, this study is the first series to investigate the relationship between plasma PRPC levels and hemorrhagic severity in addition to clinical outcomes in ICH patients. Our study confirmed that (1) as compared with controls, PRPC levels in the plasma of patients with ICH were increased significantly; (2) plasma PRPC levels, as a continuous variable, were independently related to hematoma volume and GCS score; (3) plasma PRPC levels, as a categorical variable, were independently associated with 90-day poor outcome after ICH; (4) plasma PRPC levels exhibited similar prognostic predictive ability, as compared to GCS score and hematoma volume. Hence, it is assumed that plasma PRPC may be a potential biomarker for assessing disease severity and predicting poor outcomes in patients with hemorrhagic stroke.

Cellular prion protein (PRPC) is a ubiquitously expressed plasma membrane glycoprotein (14). Accumulating experimental data have shown that PRPC may exert neuroprotective effects *via* various forms, including anti-inflammatory and anti-oxidative stress, promoting neurogenesis and angiogenesis, mediating cell protection signaling pathways, and maintaining the integrity of the blood–brain barrier after acute brain injury (15–23). PRPC expressions were significantly elevated in the ischemic hemisphere of mice with permanent focal ischemia (24). Specifically, the expression of PRPC was mainly enhanced in the cell bodies of neurons, microvascular endothelial cells, and inflammatory cells of brain tissue around the ischemic infarction (8). In patients with acute stroke, PRPC expressions were also up-regulated substantially in peri-infarcted brain tissues (25). Presumably, due to its neuroprotective characteristics, up-regulation of PRPC expressions in injured brain tissues may be a supplement against brain damage.

Although a pilot study of 37 controls and 20 head trauma patients showed an insignificant elevation of plasma PRPC levels (26), other studies on acute ischemic stroke and aneurysmal subarachnoid hemorrhage have found similar results that plasma PRPC levels were substantially raised after acute brain injury (12, 27). In line with the two preceding studies (12, 27), the current study was supportive of the notion that circulating PRPC levels should be enhanced markedly after acute brain injury. It has been verified that PRPC was greatly expressed in surrounding brain tissues after acute ischemic stroke in humans or animals (27, 28). Hence, it is assumed that PRPC in the peripheral blood may be at least partly derived from damaged brain tissues.

In patients with ischemic infarction, plasma PRPC levels are highly correlated with NIHSS scores (27). Meanwhile, in the aneurysmal subarachnoid hemorrhage patients, plasma PRPC levels were intimately related to the World Federation of Neurological Surgeons scale score, GCS score, Hunt-Hess score, and modified Fisher score (12). The above-mentioned results indicate that plasma PRPC may be a potential biomarker for reflecting the degree of brain injury. However, only univariate correlation analysis was done in the preceding epidemiological investigations. Intriguingly, using the multivariate linear regression model, we found that plasma PRPC levels were independently correlated with GCS score and hematoma volume after ICH. So, our study further supplies solid evidence supporting the notion that plasma PRPC should have the potential to serve as a promising biomarker for severity assessment after ICH. PRPC is identified as a ubiquitously expressed plasma membrane glycoprotein (14), and therefore may be released when neurons are damaged. Overall, it is assumed that PRPC may reflect the degree of neuronal injury, and plasma PRPC levels may be related to the extent of cellular membrane disruption by the form of cellular insult.

In patients with aneurysmal subarachnoid hemorrhage, plasma PRPC emerged as an independent predictor for 90-day poor outcome, but not for delayed cerebral infarction (12). Our study defined GOS scores 1–3 at 90 days after ICH as a poor outcome. Besides GCS score and hematoma volume, plasma PRPC levels were revealed to be independently associated with 90-day poor outcomes in the current study. Of note, plasma PRPC levels displayed similar prognostic predictive ability, as compared to GCS score and hematoma volume. Overall, plasma PRPC may be a useful prognostic biomarker for ICH.

## Conclusion

This is the first study to investigate the role of plasma PRPC levels in the prognosis of ICH patients and further demonstrate that elevated plasma PRPC levels, in close correlation with illness severity reflected by GCS score and hematoma volume, are independently associated with 90-day poor outcome after ICH. The preceding data substantialize plasma PRPC as a promising prognostic biomarker of ICH.

## Limitation

The current study has several limitations. First, this is a single-center study, which is characterized by small sample size, and therefore, a further cohort study with a larger sample size is required to prove the current conclusions. Second, plasma PRPC levels were only tested at the admission time. Hence, investigation of its dynamic change may be of clinical significance. Finally, in this study, there were significant differences in terms of comorbid status (hypertension, diabetes mellitus, and hyperlipidemia) between controls and patients with ICH. So, this sort of heterogeneity may lead to false-positive errors in changes in plasma PRPC levels after ICH. In the future, a control group with well-balanced comorbid status should be selected to validate the results.

## Data Availability Statement

The raw data supporting the conclusions of this article will be made available by the authors, without undue reservation.

## Ethics Statement

The studies involving human participants were reviewed and approved by Affiliated Hangzhou First People's Hospital, Zhejiang University School of Medicine. The patients/participants provided their written informed consent to participate in this study.

## Author Contributions

XW and ML contributed to the conception and design of the study, analysis and interpretation of the data, as well as drafting, and revision of the manuscript. XD and KW contributed to the analysis of the data, revision of the manuscript, and final approval of the manuscript. TY and ZW participated in the study design and performed the follow-up. WY, QD, WH, ZZ, and YZ helped to sort data. All authors contributed to the article and approved the submitted version.

## Funding

This work is financially supported by Key Research and Development Projects of Zhejiang Province (Grant. 2020C03071) and Construction Fund of Medical Key Disciplines of Hangzhou (Nos. OO20200485 and OO20200055).

## Conflict of Interest

The authors declare that the research was conducted in the absence of any commercial or financial relationships that could be construed as a potential conflict of interest.

## Publisher's Note

All claims expressed in this article are solely those of the authors and do not necessarily represent those of their affiliated organizations, or those of the publisher, the editors and the reviewers. Any product that may be evaluated in this article, or claim that may be made by its manufacturer, is not guaranteed or endorsed by the publisher.
